# More than credit: Exploring associations between microcredit programs and maternal and reproductive health service utilization in India

**DOI:** 10.1016/j.ssmph.2019.100467

**Published:** 2019-08-06

**Authors:** Nabamallika Dehingia, Abhishek Singh, Anita Raj, Lotus McDougal

**Affiliations:** a Center on Gender Equity and Health, Department of Medicine, University of California San Diego, La Jolla, CA, USA; b Joint Doctoral Program, San Diego State University/University of California San Diego, CA, USA; c Department of Public Health & Mortality Studies, International Institute for Population Sciences, Mumbai, India

**Keywords:** Microcredit program, Financial inclusion, India, Maternal health, Postpartum contraceptive

## Abstract

Microcredit programs are increasingly popular interventions aimed at enabling women's economic empowerment in low- and middle-income countries. Resultant improved income, and social support from co-members of microcredit programs, may lead to increased utilization of health services. But existing research is inconclusive. This study investigates the association of microcredit program awareness and participation, with maternal and postpartum reproductive health service utilization in India. We use data from a nationally representative survey, the National Family Health Survey (2015–16), and assess three indicators of maternal health service utilization: receipt of four or more antenatal check-ups, institutional delivery, and postnatal check-up among women who had a child less than 5 years of age (N = 32,880). Reproductive health service utilization is assessed via postpartum contraceptive use within 12 months of childbirth, among women who had a live birth in the 12–59 months preceding the survey (N = 24,258). We use binomial and multinomial logistic regression models to examine associations. Additionally, we use propensity score matching to account for self-selection bias. One-third of women are aware of microcredit programs in their community/village, but only 6% have ever taken a loan from these programs. Both microcredit program awareness and participation are associated with higher odds of antenatal care, postnatal check-ups, as well as use of a modern method of contraceptive within 12 months of childbirth, even after accounting for self-selection bias. Stratified analysis by household wealth show that significant associations seen in our primary analyses are significant only for the poorest women. Findings highlight the potential value of microcredit programs in improving health service utilization during and after pregnancy, particularly among poor women. Microcredit program benefits extend beyond their participants. Non-participants living close to the programs also have greater odds of maternal and reproductive health service utilization, suggesting a spillover effect of these programs at the community level.

## Highlights

• Microcredit program awareness and participation are associated with improved maternal and postpartum reproductive health service utilization.
• Observed associations are true even after accounting for self-selection bias.
• Observed associations are strongest for women belonging to the lowest wealth quintile.

## Introduction

Microcredit programs provide micro-loans to low-income individuals, to address household financial needs or entrepreneurship, enabling them to achieve financial security. Over the last 20 years, these programs have been a key poverty reduction strategy globally (S.B. Banerjee & Jackson, 2017), with most programs targeting women as clients (D'espallier, Guerin, & Mersland, 2013; Siraj, 2012). A growing body of research suggests that through access to credit, microcredit programs benefit women in the form of increased household income (Awunyo-Vitor, Abankwah, & Kwansah, 2012; Nudamatiya, Giroh, & Shehu, 2010), poverty alleviation (Khandker, 2005), better schooling for their children (Holvoet, 2004) and improved status of women within their households and communities (Norwood, 2014; Panjaitan-Drioadisuryo & Cloud, 1999).

Recent studies have also explored the health effects of microcredit programs in low and middle-income countries, particularly with respect to maternal and reproductive health (Somen Saha, Annear, & Pathak, 2013; Steele, Amin, & Naved, 2001). Existing literature suggests several pathways through which access to credit may lead to improved utilization of health services. With increasing income, a household may have greater ability to pay for, and access healthcare (Braveman, Cubbin, Egerter, Williams, & Pamuk, 2010). Microcredit programs that follow the group-lending model can cause an improvement in social support and networking, leading to an increased spread of knowledge regarding health services (Ruducha et al., 2018). Women's economic inclusion through microcredit programs can also facilitate a growing awareness of the value of healthcare and a sense of self-efficacy to prioritize their health (Cornwall, 2016). A longitudinal study in Bangladesh found positive associations between membership in a microcredit program and contraceptive use, even after accounting for selection bias among members (Steele et al., 2001). Findings from rural India indicate improved contraceptive use, institutional delivery rate, and newborn care among members of the programs (Somen Saha et al., 2013). The effects of microcredit programs, however, are not uniformly positive across different contexts. A randomized control trial in Ethiopia found no change in contraceptive use among the communities receiving the microcredit intervention (Desai & Tarozzi, 2011). Another study from Peru reported absence of any relationship between length of participation in a microcredit program and contraception use (Hamad & Fernald, 2015). Few studies have pointed to women entering a cycle of debt as a result of microcredit program participation (Rahman, 1999), which might explain the absence of, or negative effects of microcredit programs on its members. The study by Rahman found that a process of loan recycling increases stress and frustration among the household members of the women, eventually leading to increased violence against women. Researchers have also argued that microcredit programs are mostly shaped by local structures of power. In India, caste, the marker of one's social status, and economic status, have been observed to be the key actors in microcredit program implementation in local communities; dominant caste and class groups benefit most from the programs (Guerin & Kumar, 2017).

The mixed findings with regards to association between microcredit programs and indicators of health suggest the unique experience of the programs in different contexts and geographies, making generalization of studies difficult. This is not surprising, given that different models of microcredit programs are implemented in different settings. The group-lending, or Self-Help Groups (SHG) model is the most popular form of microcredit programs, which includes frequent interactions between members and entail a strong commitment towards solving members’ problems through mutual aid and support, creating solidarity and social capital (Folgheraiter & Pasini, 2009). SHGs integrated with different health promotion activities is another model being implemented in many African and South Asian countries (Gugerty, Biscaye, & Leigh Anderson, 2019). Different from the SHG model is financial institutions providing collateral free or low-interest microloans to poor individuals. Such programs usually include a broad range of financial inclusion related services such as bank account ownership, bank transfers and micro-insurance (Batra, 2012). In addition to the presence of varying models, microcredit programs might also exhibit differential relationship with different health outcomes. For instance, one can hypothesize that microcredit program participation is associated with use of contraceptives post child birth, with the assumption that an increase in income can provide a couple with the financial ability to buy contraceptives. However, reproductive health behavior such as desiring a child after the woman turns 18 might need interventions aimed to change social norms, and economic approaches such as microcredit programs might not be very effective.

India presents an interesting setting in which to test the relationship between microcredit programs and women's health. Microcredit programs have existed in the country since the early 1980s, and they do not follow a single model. The programs have been implemented by different agencies in different regions, including the government, not-for-profit private organizations and civil society organizations. The different models include the financial institutions which provide interest-free loans, and SHGs. However, the majority of the programs today follow the SHG model (Batra, 2012; Sankaran, 2005). Most SHGs in India have evolved to act as more than just a conduit for credit and social support; they also act as delivery mechanisms for various other services such as entrepreneurial and leadership training, and health promotion interventions. The health-based SHGs usually include participatory behavior communication on maternal, neonatal, child health (Saggurti et al., 2018). These interventions do not usually have any formal intersection with the health systems. Instead they aim to promote correct health behavior within the participating women. Studies examining the health effects of SHGs in India have found positive association with antenatal care, contraceptive use and institutional deliveries (Saggurti et al., 2018; S.; Saha, Kermode, & Annear, 2015). Most of the research on microcredit programs in India has been evaluation studies for specific interventions using SHGs as a platform for service provision. Nationally representative studies of microcredit programs, examining the relationship between economic participation of women and health, are rare. Additionally, studies assessing presence of a program near women, measured via their awareness of a program, are uncommon. By living close to women who participate in microcredit programs, non-participants might benefit by receiving new information from participants.

In addition to the existing gaps in literature with regards to research on microcredit programs, another reason that makes this study important is its focus on maternal and reproductive health services in India. Utilization of maternal health services has seen a substantial increase in the recent years, with great progress in reducing maternal mortality from 556 per 100 000 live births in 1990 to 130 per 100 000 live births in 2016 (WHO, 2019b). India launched National Rural Health Mission (NRHM) in 2005, now expanded to be known as National Health Mission (NHM), to improve the overall health of women and children in the country. Its strategies target both access-related and behavioral challenges, such as, recruitment of community health workers to provide women with necessary information on antenatal care, postnatal care, and contraceptives, provision of financial incentives to poor women for institutional delivery, and ensuring quality of services provided at health facilities during childbirth. Population level studies have shown significant increase in key maternal and child health services such as antenatal care, institutional delivery and immunizations, since NRHM was set up. However, indicators such as use of modern contraceptives among married women, and inequities with respect to health outcomes have shown less improvement (Vellakkal et al., 2017). In this context, examining the contribution of non-health related interventions, such as microcredit programs to improving the health status of women in India is relevant. Such research can contribute to designing innovative interventions that can accelerate India's growth towards universal health coverage. Studies on microcredit programs and health in India have focused on a limited number of individual outcomes related to maternal and reproductive health. By looking at service utilization across the continuum of antenatal, intrapartum as well as post-partum care, in a nationally representative sample of women, our study aims to present a comprehensive understanding of effects of microcredit programs on women's health during, as well as post pregnancy.

The current study aims to assess the association of participation in microcredit programs, and awareness of these programs, with maternal and reproductive health service utilization, among women in India. We hypothesize that women who have ever participated in a microcredit program in her community/village by taking loans, are more likely to have used maternal and reproductive health services during their most recent episode of childbirth, as compared to those who have never participated in such programs. Our second hypothesis is that women who are aware of any microcredit program in her community/village, or live near a microcredit program, are more likely to be using maternal and reproductive health services, as compared to those who are not aware. Our findings are expected to contribute to the discourse on the use of economic approaches for improving women's health.

## Methods

We used data from the National Family Health Survey (NFHS-4), a nationally representative Indian household survey. The survey was conducted from 2015-2016 and interviewed women of 15–49 years on a number of areas including their socio-demographic characteristics and health behaviors; survey design has been described elsewhere (International Institute Population Sciences and ICF). A sub-sample of women were asked questions on employment, financial engagement, freedom of movement and decision-making power within the household. This analysis includes the women from this sub-sample who had their most recent live birth in the 5 years (0–59 months) preceding the survey (N = 32,880). The analysis on postpartum contraceptive use includes women who had a live birth during 12 to59 months preceding the survey, to allow the 12 month-window for post-partum contraceptive use (N = 24,258).

### Measures

We assessed three indicators of maternal health service utilization: receipt of minimum 4 antenatal care (ANC) visits during pregnancy (based on current WHO standard for adequate ANC) (WHO, 2017), institutional delivery and postnatal care. We used one indicator to assess reproductive health service utilization: postpartum contraceptive use. Institutional delivery assessed whether women delivered at any government health facility, privately owned hospital/clinic or an NGO hospital/clinic for the most recent childbirth. Postnatal care referred to receipt of any check-up by women, within 48 h of childbirth. Women who used any contraceptive method within 12 months after childbirth were identified as having used postpartum contraception, following WHO recommendation (WHO, 2013). Based on the first method used after childbirth, women were then categorized as traditional vs. modern method users. Traditional methods included withdrawal and rhythm method. Modern methods included female and male sterilization, injectables, implants, IUD, pills, condoms, female condoms, foam, lactational amenorrhea and standard days method (based on WHO definitions) (WHO, 2019a).

The predictor variable for the current analysis was microcredit program awareness and participation. Women were asked if they ‘*knew of any programs in [their] area that give loans to women to start or expand a business of their own*’ and whether they had ‘*ever taken any loan from those programs to start or expand a business*’. Based on these two questions, we computed the microcredit program variable, with three categories: not aware of any microcredit program, aware of a program but never taken any loans, taken loans from a microcredit program. For an additional analysis to examine association between living near a microcredit program and the outcomes, we created a variable for nearness to microcredit programs, with three categories - women who resided in the same cluster as a women participating in microcredit program but never herself took any loans, women who took loans from a microcredit program, and women who neither took loans nor lived in the same area as the participating women. The study defined cluster as villages (rural areas) or census enumeration blocks (urban areas); the cluster variable was used to identify those women who reported to not have participated in a microcredit program but lived near anyone who was a participant.

We grouped the covariates into (a) index pregnancy-related variables, (b) variables related to gender equity and (c) socio-demographic variables. Covariates related to index pregnancy-related variables included parity (categorized as 1, 2, 3 + births) and any son born before the index pregnancy (Yes/No). For the models with postpartum contraceptive use as outcome, the covariate ‘any son born’ included the gender of the index pregnancy outcome as well, to account for India's known history of son preference (Bandyopadhyay & Singh, 2003). The second group of variables related to gender equity were included to adjust for existing gender norms in India. These norms often dictate freedom of independent mobility for women, having access to financial resources, and when and whom a woman should get married to – all of which have shown to have adverse effects on her maternal and reproductive health outcomes (Bloom, Wypij, & Gupta, 2001; Chol, Negin, Agho, & Cumming, 2019). The group of variables related to gender equity thus included four variables: having own money that women alone can decide how to use (yes/no), freedom of movement, child marriage and age gap between women and her husband (categorized as less than 5 yrs, 5–12 yrs, more than 12 yrs). Women were asked if they were allowed to travel alone to places outside her village, to a market and to a health facility. A positive response for all the three items was categorized as having ‘freedom of movement’. Child marriage assessed if women were married before the age of 18.

Sociodemographic items on the woman included maternal age, maternal schooling (categorized as less than 5 yrs, 5–12 yrs, more than 12 yrs), caste (a marker of social status in Indian society and categorized as Scheduled Caste/Scheduled Tribe- SC/ST, Other Backward Class- OBC, and Other Caste- OC), religion (dichotomized as Muslim and non-Muslim), household wealth, bank account ownership (yes/no), frequent access to information, region (place of residence) and rural/urban (dichotomous variable). The variable on caste has been added owing to numerous studies indicating the vast inequalities that exist in India based on this indicator; those belonging to SC/ST and OBC have adverse mortality as well as morbidity outcomes as compared to those belonging to General or OC (Jungari & Chauhan, 2017). A composite measure of a household's cumulative living standard, wealth index, was provided with the data, and calculated using principal component analysis on variables capturing household's ownership of selected assets. This continuous index of relative wealth was divided into five wealth quintiles, which constituted the household wealth variable. Frequent access to information assessed whether or not women read a newspaper, or watched television, or listened to the radio almost every day. Region assessed the state in which women were residing at the time of the survey. It was categorized as North (Chandigarh, Delhi, Haryana, Himachal Pradesh, Jammu and Kashmir, Punjab, Rajasthan, Uttarakhand), Central (Chhattisgarh, Madhya Pradesh, Uttar Pradesh), East (Bihar, Jharkhand, Odisha, West Bengal), Northeast (Arunachal Pradesh, Assam, Manipur, Meghalaya, Mizoram, Nagaland, Sikkim, Tripura), West (Dadra and Nagar Haveli, Daman and Diu, Goa, Gujarat, Maharashtra) and South (Andaman and Nicobar Islands, Andhra Pradesh, Karnataka, Kerala, Lakshadweep, Puducherry, Tamil Nadu, Telangana).

### Statistical analysis

Descriptive analyses assessed the prevalence of outcomes as well as various predictors. We used three adjusted binomial logistic regression models to determine whether microcredit program awareness and participation were associated with the three indicators of maternal health service utilization. No multicollinearity was present in the multivariate regression models using a variance inflation factor cut-off of five (Stine, 1995).

We used two separate multinomial logistic regression models to examine postpartum reproductive health service utilization: (a) outcome categorized as women who did not use any method, women who used any traditional method and women who used any modern method (b) outcome categorized as women who used any limiting method (female and male sterilization), women who used any modern spacing method, women who used any traditional method and women who used no method.

The five adjusted regression models specified above were also run with nearness to a microcredit program as the key predictor, instead of the variable on microcredit program awareness and participation, to examine the association between living close to microcredit programs and health.

We used three limited-sample regression models, one for each of the three outcomes of maternal health service utilization. These models included the sub-sample of women who had at least one live birth in the 12 months preceding the survey, as opposed to live births in 5 years preceding the survey in the original models. We carried out these tests to assess a potential temporal relationship between microcredit programs and maternal health services. The models were adjusted for all the covariates. We also used five limited-sample regression models, one each for the wealth quintiles to assess any differential impact of microcredit programs by wealth status. These models were adjusted for all the covariates. To account for any self-selection bias in microcredit program awareness and participation, we used propensity score matching (PSM) using inverse probability of treatment weighting (IPTW) technique (McCaffrey et al., 2013) to balance the three groups of the variable microcredit awareness and participation. The observations were matched at the individual, i.e. women level. IPTW is based on the assumption that the different groups differ in their distributions of pretreatment variables and, therefore, possibly differ in terms of their observed outcomes in ways that are not attributable to treatment. IPTW reweights a treatment sample to make the distribution of the pretreatment variables match that of any of the other treatment groups. Using the *twang* package in R, which allows for multinomial propensity score generation (more than two groups in the treatment variable), we generated the weights (Ridgeway, McCaffrey, Morral, Burgette, & Griffin, 2017). We estimated the average treatment effect (ATE) which is the average effect, at the population level, of moving an entire population from untreated to treated and the average treatment on treated (ATT), which is the average effect of treatment on those subjects who ultimately received the treatment. We assessed two treatments, awareness of microcredit program and participation in microcredit program. Women who were not aware of and had never participated in a microcredit program formed the reference or control category. We used chi-square tests to identify the covariates to be included in the PSM model; all the covariates which showed significant differences (significance level 5%) between groups were included.

Analyses were conducted using Stata SE 15 and R version 5.3.1 and adjusted for survey design and sampling weights.

## Results

Most women in the sample (82.2%) had an institutional delivery and received a postnatal check-up (66.3%), but only just over half received the required minimum four ANC visits during their pregnancy (54.4%) (Table 1). Among those who had a live birth in 12–59 months preceding the survey, nearly one-half (47.7%) started using a contraceptive method within 12 months postpartum. Among modern contraceptive users, the most prevalent method was female sterilization, followed by condoms. Nearly one in three (31.8%) women in the sample were aware of the presence of any microcredit program in their community; only 6.4% reported ever having taken a loan from the programs. Fifty-nine percent had frequent access to information via TV, newspapers or radio and 40% had their own money that they could spend. Around one-third of the women were married before 18 years of age and the mean age of the women was 28 years. Around six percent of women had an age gap of more than 10 years with their husbands. Chi-square tests showed significant associations between each individual covariate and the four outcomes, except religion and receipt of minimum four ANC.

**Table 1:** Sample characteristics.

Variables	Total	Receipt of minimum 4 ANC			Institutional delivery			Postnatal care			Postpartum contraceptive			
		N = 32,880			N = 32,880			N = 32,880			N = 24,258			
		*No*	*Yes*		*No*	*Yes*		*No*	*Yes*		*No method*	*Traditional method*	*Modern method*	
		(wtd%= 45.59%)	(wtd%= 54.41%)		(wtd%= 17.78%)	(wtd%= 82.22%)		(wtd%= 33.66%)	(wtd%= 66.34)		(wtd%= 52.32%)	(wtd%= 9.10%)	(wtd%= 38.58%)	
	Wtd. %/mean	Wtd. %/mean	Wtd. %/mean	p-value*	Wtd. %/mean	Wtd. %/mean	p-value*	Wtd. %/mean	Wtd. %/mean	p-value*	Wtd. %/mean	Wtd. %/mean	Wtd. %/mean	p-value*
Microcredit program														
Does not know of any microcredit program	61.72	71.31	53.68	0.00	72.46	59.40	0.00	72.37	56.32	0.00	65.95	57.36	55.52	0.00
Aware of a program	31.84	24.00	38.41		22.90	33.78		22.59	36.54		27.43	37.16	36.29	
Participated in a program	6.44	4.69	7.90		4.64	6.82		5.044	7.142		6.623	5.481	8.192	
Index-pregnancy related variables														
Birth parity														
1	33.85	26.37	40.12	0.00	15.46	37.83	0.00	27.05	37.3	0.00	38.1	38.33	22.79	0.00
2	34.62	29.99	38.49		28.15	36.01		31.58	36.16		29.46	32.02	42.81	
3+	31.53	43.64	21.39		56.40	26.16		41.37	26.54		32.43	29.65	34.4	
Any son born before the index birth														
Yes	38.62	53.04	68.36	0.00	41.28	65.73	0.00	53.52	65.36	0.00	64.14	64.89	51.99	0.00
No	61.38	46.96	31.64		58.72	34.27		46.48	34.64		35.86	35.11	48.01	
Gender equity														
Having own money														
Yes	39.9	63.94	56.88	0.00	63.85	59.29	0.00	65.04	57.59	0.00	62.38	52.87	55.47	0.00
No	60.1	36.06	43.12		36.15	40.71		34.96	42.41		37.62	47.13	44.53	
Freedom of movement														
Yes	35.32	69.63	60.54	0.00	68.83	63.79	0.00	68.42	62.79	0.00	66.14	60.87	58.09	0.00
No	64.68	30.37	39.46		31.17	36.21		31.58	37.21		33.86	39.13	41.91	
Child marriage														
Yes	35.78	57.35	69.99	0.00	50.03	67.29	0.00	57.4	67.69	0.00	62.54	66.14	63.35	0.08
*No*	64.22	42.65	30.01		49.97	32.71		42.6	32.31		37.46	33.86	36.65	
Age gap between partners														
0–4 years	68.15	73.86	63.36	0.00	74.27	66.82	0.00	70.73	66.84	0.00	69.19	68.25	64.35	0.00
5–10 years	25.46	20.79	29.37		20.76	26.48		22.98	26.72		24.48	25.18	27.91	
more than 10	6.39	5.34	7.27		4.96	6.70		6.291	6.443		6.328	6.561	7.742	
Socio-demographics														
Education														
0–4 years	31.8	46.34	19.62	0.00	60.87	25.51	0.00	43.49	25.87	0.00	36.49	30.37	27.15	0.00
5–12 years	55.21	46.88	62.20		37.04	59.14		48.7	58.51		51.27	54.9	59.06	
more than 12	12.99	6.79	18.18		2.09	15.34		7.805	15.62		12.24	14.73	13.79	
Religion														
Muslim	16.36	83.26	83.96	0.25	76.54	85.18	0.00	81.58	84.69	0.00	82.85	83.06	84.26	0.17
Hindu	83.64	16.74	16.04		23.46	14.82		18.42	15.31		17.15	16.94	15.74	
Caste														
SC/ST	29.85	19.58	30.09	0.00	19.72	26.50	0.00	22.51	26.71	0.00	22.67	32.75	29.14	0.00
OBC	44.85	47.85	42.35		41.82	45.51		44.64	44.96		48.04	38.1	41.79	
Other	25.3	32.58	27.56		38.46	27.99		32.85	28.33		29.29	29.15	29.07	
*Age of woman (years; continuous)*	26.98	27.39	26.65	0.00	28.23	26.72	0.00	27.35	26.80	0.00	27.58	27.84	27.71	0.06
Wealth quintile														
1	21.87	34.90	10.96	0.00	47.51	16.33	0.00	33.17	16.14	0.00	26.15	19.99	15.44	0.00
2	20.63	24.72	17.20		25.75	19.52		23.92	18.95		20.57	20.59	20.1	
3	20.42	17.99	22.45		14.77	21.64		18.42	21.43		19.27	19.46	21.98	
4	18.97	12.90	24.05		8.41	21.25		14.18	21.4		18.14	17.78	20.93	
5	18.12	9.50	25.34		3.56	21.27		10.31	22.08		15.87	22.18	21.55	
Bank account														
No bank account	51.34	58.82	45.07	0.00	66.25	48.11	0.00	59.06	47.42	0.00	54.08	46.97	47.05	0.00
Has a bank account	48.66	41.18	54.93		33.75	51.89		40.94	52.58		45.92	53.03	52.95	
Access to information														
Yes	58.67	59.27	26.30	0.00	69.91	35.15	0.00	54.85	34.47	0.00	47.12	41.15	31.55	0.00
No	41.33	40.73	73.70		30.09	64.85		45.15	65.53		52.88	58.85	68.45	
Region														
North	13.05	12.92	13.16	0.00	10.50	13.60	0.00	11.26	13.96	0.00	9.792	18.11	16	0.00
West	15.02	8.69	20.32		7.74	16.59		11.18	16.96		14.83	10.77	16.19	
South	20.36	9.20	29.72		3.50	24.01		13.76	23.71		21.74	4.777	23.14	
Northeast	3.62	3.92	3.37		5.70	3.17		4.541	3.153		3.014	7.371	3.906	
East	24.14	30.03	19.21		37.18	21.32		30.87	20.73		25.33	26.77	21.83	
Central	23.81	35.24	14.23		35.38	21.31		28.39	21.49		25.29	32.2	18.93	
Rural/Urban														
Rural	69.01	21.49	38.96	0.00	16.72	34.08	0.00	23.51	34.79	0.00	29.21	33.84	34.17	0.00
Urban	30.99	78.51	61.04		83.28	65.92		76.49	65.21		70.79	66.16	65.83	

*p-values are for chi-square analysis for the outcome variable and categorical variables, and F-tests for the outcome variable and continuous variable ‘age of woman’.

Both microcredit program awareness and participation were associated with each individual covariate except age of the woman (Table 2). Women with more years of education, with bank account ownership, with frequent access to information and belonging to higher wealth quintile were more likely to be aware of, or to have participated in, a microcredit program.

**Table 2:** Sample characteristics by microcredit program awareness and participation (N = 32,880).

Variables	Total	Not aware of a microcredit program (wtd. % = 61.72%)	Aware of a microcredit program (wtd. % = 31.84%)	Participated in a microcredit program (wtd. % = 6.44%)	
	Wtd. %/mean	Wtd. %/mean	Wtd. %/mean	Wtd. %/mean	p-value^a^
Index-pregnancy related variables					
Birth parity					
1	33.85	58.63	35.88	5.48	0.000
2	34.62	59.01	33.48	7.51	
3+	31.53	68.01	25.71	6.28	
Any son born before the index birth					
Yes	38.62	65.51	27.84	6.65	0.000
No	61.38	59.34	34.36	6.30	
Gender equity					
Having own money					
Yes	39.90	55.48	37.48	7.04	0.000
No	60.10	65.86	28.10	6.03	
Freedom of movement					
Yes	35.32	56.47	34.69	8.38	0.000
No	64.68	64.58	30.29	5.12	
Child marriage					
Yes	35.78	65.64	27.51	6.84	0.000
*No*	64.22	59.53	34.26	6.21	
Age gap between partners					
0–4 years	68.15	64.22	30.31	5.46	0.000
5–10 years	25.46	56.75	35.08	8.17	
more than 10	6.39	54.82	35.26	9.92	
Socio-demographics					
Education					
0–4 years	31.80	72.35	22.27	5.38	0.000
5–12 years	55.21	58.33	34.61	7.06	
more than 12	12.99	50.11	43.51	6.38	
Religion					
Muslim	16.36	65.50	29.02	5.47	0.004
Hindu	83.64	60.98	32.4	6.63	
Caste					
SC/ST	29.85	62.31	30.26	7.43	0.000
OBC	44.85	62.45	31.18	6.37	
Other	25.3	59.73	34.89	5.38	
*Age of woman (years; continuous)*	26.98	26.98	26.86	27.64	0.065
Wealth quintile					
1	21.87	73.63	21.51	4.86	0.000
2	20.63	65.77	28.45	5.79	
3	20.42	58.87	32.75	8.38	
4	18.97	54.54	37.37	8.09	
5	18.12	53.47	41.38	5.15	
Bank account					
No bank account	51.34	69.63	26.82	3.55	0.000
Has a bank account	48.66	53.37	37.15	9.48	
Access to information					
Yes	58.67	54.55	37.70	7.75	0.000
No	41.33	71.90	23.53	4.57	
Region					
North	13.05	70.76	27.69	1.55	0.000
West	15.02	63.31	32.90	3.79	
South	20.36	43.68	41.59	14.73	
Northeast	3.62	62.75	31.12	6.13	
East	24.14	61.66	30.81	7.54	
Central	23.81	71.10	26.28	2.62	
Rural/Urban					
Rural	69.01	64.61	29.26	6.14	0.000
Urban	30.99	55.29	37.61	7.1	

a p-values - chi-square analysis for microcredit program awareness and participation, and individual covariates.

### Maternal health service utilization

Awareness of microcredit programs was associated with the three indicators of maternal health service utilization (AOR 1.51; 95% CI: 1.40–1.64 for ANC, AOR 1.16; 95% CI: 1.05–1.28 for institutional delivery, AOR 1.70; 95% CI: 1.57–1.84 for postnatal care) (Table 3). Participation in microcredit programs was also associated with ANC (AOR 1.41; 95% CI: 1.14–1.74) and postnatal care (AOR: 1.54; 95% CI: 1.30–1.82). Years of education, ownership of a bank account, frequent access to information and increasing wealth status were significantly, and positively associated with receipt of minimum four ANC, institutional delivery and postnatal check-up. Higher parity and presence of a living son reduced the odds of all three indicators of maternal health in the adjusted models. Women residing in the ‘South’ region of the country showed increased likelihood of all three indicators of maternal health.

**Table 3:** Logistic regression models assessing predictors of maternal health services – receipt of ANC, institutional delivery and postnatal care (N = 32,880).

	Receipt of minimum 4 ANC		Institutional delivery		Postnatal care	
Independent variables	Unadjusted OR	Adjusted OR	Unadjusted OR	Adjusted OR	Unadjusted OR	Adjusted OR
*Microcredit program (REF:* Does not know of any microcredit prog)						
Aware of a program	2.13***	1.51***	1.80***	1.16***	2.08***	1.70***
	(1.98–2.28)	(1.40–1.64)	(1.65–1.97)	(1.05–1.28)	(1.93–2.24)	(1.57–1.84)
Participated in a program	2.24***	1.41***	1.79***	1.13	1.82***	1.54***
	(1.91–2.63)	(1.14–1.74)	(1.47–2.19)	(0.91–1.40)	(1.55–2.13)	(1.30–1.82)
Index-pregnancy related variables						
Parity (REF: 1)						
Parity (2)	0.84***	0.92	0.52***	0.59***	0.83***	0.92*
	(0.78–0.91)	(0.83–1.03)	(0.46–0.59)	(0.51–0.68)	(0.76–0.90)	(0.83–1.01)
Parity (3 or more)	0.32***	0.62***	0.19***	0.43***	0.47***	0.77***
	(0.30–0.35)	(0.54–0.70)	(0.17–0.21)	(0.37–0.50)	(0.43–0.50)	(0.68–0.86)
Any son born before the index birth (REF: No)						
Yes	0.52***	0.89***	0.37***	0.80***	0.61***	0.88***
	(0.49–0.56)	(0.81–0.97)	(0.34–0.40)	(0.72–0.88)	(0.57–0.65)	(0.81–0.96)
Gender equity						
Has own money (REF: No)						
	1.34***	1.08**	1.21***	0.94	1.37***	1.130***
	(1.26–1.43)	(1.00–1.17)	(1.12–1.31)	(0.86–1.03)	(1.28–1.47)	(1.05–1.22)
Freedom of movement (REF: No)						
Yes	1.49***	1.21***	1.25***	1.06	1.28***	1.10**
	(1.40–1.60)	(1.12–1.31)	(1.15–1.36)	(0.97–1.17)	(1.20–1.38)	(1.02–1.19)
Child marriage (REF: No)						
Yes	0.58***	0.95	0.48***	0.88***	0.64***	0.90***
	(0.54–0.62)	(0.88–1.03)	(0.45–0.52)	(0.80–0.96)	(0.61–0.68)	(0.83–0.97)
Age gap between partners (REF: less than 5)						
5–10 years	1.65***	1.16***	1.42***	1.03	1.23***	1.05
	(1.52–1.78)	(1.06–1.27)	(1.29–1.56)	(0.92–1.145)	(1.14–1.33)	(0.97–1.14)
more than 10 years	1.59***	1.16*	1.50***	1.20*	1.08	0.96
	(1.39–1.81)	(0.99–1.35)	(1.25–1.80)	(0.99–1.46)	(0.94–1.24)	(0.83–1.11)
Socio-demographics						
Education years (REF: less than 5)						
5–12 years	3.13***	1.36***	3.81***	1.51***	2.02***	1.12***
	(2.92–3.36)	(1.25–1.48)	(3.52–4.13)	(1.36–1.67)	(1.89–2.16)	(1.04–1.22)
more than 12 years	6.33***	1.57***	17.53***	2.69***	3.36***	1.15*
	(5.63–7.11)	(1.35–1.83)	(13.89–22.11)	(2.06–3.50)	(2.95–3.84)	(0.98–1.34)
Religion (REF: Hindu/Other)						
Muslim	0.95	1.20***	0.57***	0.59***	0.80***	0.90**
	(0.87–1.04)	(1.07–1.34)	(0.51–0.63)	(0.52–0.66)	(0.73–0.88)	(0.81–0.99)
Caste (REF: Other)						
Caste (OBC)	0.58***	0.71***	0.81***	0.91	0.85***	0.98
	*(0.53 - 0.63)*	*(0.63 - 0.78)*	(0.72–0.90)	(0.80–1.04)	(0.78–0.93)	(0.89–1.08)
Caste (SC/ST)	0.55***	0.96	0.54***	0.75***	0.73***	0.99
	(0.50–0.60)	(0.86–1.08)	(0.48–0.61)	(0.66–0.86)	(0.66–0.80)	(0.89–1.10)
Age of woman (continuous)	0.97***	1.00	0.95***	0.99	0.98***	0.99
	(0.97–0.98)	(0.99–1.01)	(0.94–0.95)	(0.99–1.01)	(0.97–0.99)	(0.99–1.003)
Wealth (REF: 1/Poorest)						
Wealth (2)	2.22***	1.38***	2.21***	1.44***	1.63***	1.274***
	(2.01–2.44)	(1.24–1.54)	(2.00–2.43)	(1.29–1.61)	(1.49–1.78)	(1.16–1.40)
Wealth (3)	3.98***	1.65***	4.26***	1.82***	2.39***	1.49***
	*(3.60 - 4.39)*	*(1.46 - 1.87)*	*(3.81 - 4.77)*	(1.58–2.09)	*(2.18 - 2.63)*	*(1.33 - 1.67)*
Wealth (4)	5.94***	1.95***	7.36***	2.36***	3.10***	1.67***
	(5.33–6.62)	(1.69–2.25)	(6.38–8.48)	(1.96–2.85)	(2.80–3.43)	(1.46–1.91)
Wealth (5/Wealthiest)	8.50***	2.43***	17.36***	4.11***	4.40***	2.11***
	(7.53–9.58)	(2.04–2.89)	(14.40–20.93)	(3.19–5.28)	(3.88–4.98)	(1.79–2.50)
Bank account (REF: does not have a bank account)						
Has a bank account	1.74***	1.13***	2.12***	1.38***	1.60***	1.14***
	(1.63–1.85)	(1.04–1.22)	(1.96–2.29)	(1.26–1.51)	(1.50–1.71)	(1.06–1.23)
Access to information (REF: no frequent access)						
Frequent access to information	4.08***	1.60***	4.29***	1.21***	2.31***	1.18***
	(3.82–4.36)	(1.45–1.77)	(3.94–4.66)	(1.08–1.35)	(2.16–2.47)	(1.08–1.28)
Region (REF: North)						
Region (West)	2.30***	2.37***	1.65***	1.54***	1.22***	1.17**
	(2.01–2.62)	(2.05–2.74)	(1.39–1.97)	(1.27–1.86)	(1.07–1.40)	(1.02–1.35)
Region (South)	3.17***	2.54***	5.30***	3.55***	1.39***	1.07
	(2.80–3.59)	(2.21–2.93)	(4.19–6.70)	(2.77–4.54)	(1.24–1.56)	(0.94–1.22)
Region (Northeast)	0.84***	1.09	0.43***	0.59***	0.56***	0.69***
	(0.76–0.94)	(0.95–1.24)	(0.38–0.49)	(0.51–0.68)	(0.50–0.63)	(0.61–0.78)
Region (East)	0.63***	1.02	0.44***	0.77***	0.54***	0.74***
	(0.57–0.69)	(0.92–1.13)	(0.39–0.50)	(0.68–0.88)	(0.49–0.60)	(0.66–0.82)
Region (Central)	0.40***	0.61***	0.46***	0.71***	0.61***	0.81***
	(0.37–0.43)	(0.55–0.66)	(0.42–0.52)	(0.63–0.80)	(0.56–0.66)	(0.74–0.89)
Rural/Urban (REF: Urban)						
Rural	0.43***	0.97	0.39***	1.02	0.58***	0.99
	(0.40–0.46)	(0.88–1.08)	(0.35–0.43)	(0.90–1.16)	(0.53–0.63)	(0.91–1.10)

***p < 0.01, **p < 0.05, *p < 0.1.

Results from models with the sub-sample of women who had a live birth during 12 months preceding the survey indicated significant association of microcredit program awareness with ANC as well as postnatal care (AOR 1.52; 95% CI: 1.30–1.77 for ANC, AOR 1.57; 95% CI: 1.36–1.82 for postnatal care) (Supplement Table A). Microcredit program participation was also significantly associated with both ANC and postnatal care (AOR 1.92; 95% CI: 1.32–2.79 for ANC, AOR 1.61; 95% CI: 1.13–2.29 for postnatal care).

### Reproductive health service utilization

Both awareness of, and participation in a microcredit program were associated with use of any modern method of contraception post childbirth (ARRR: 1.45; 95% CI: 1.32–1.59 for microcredit awareness and ARRR: 1.30; 95% CI: 1.09–1.55 for microcredit participation) (Table 4). Women aware of a microcredit program had a greater likelihood of using a modern spacing contraception after childbirth (ARRR:1.55; 95% CI: 1.39–1.74). Significant association was also observed for microcredit program participation and use of a spacing method postpartum (ARRR: 1.34; 95% CI: 1.05–1.72), when compared to women who were not using any method. Among those who used any limiting method, 99.5% were using female sterilization.

**Table 4:** Multinomial logistic regression models assessing predictors of postpartum contraceptive use (N = 24,258).

Variables	Model 1: Multinomial model with outcome categories: no use, traditional method and modern method (Ref: no use of contraceptive)		Model 2: Multinomial model with outcome categories: no use, traditional method use, limiting method use and spacing method use (Ref: no use of contraceptive)		
	Traditional methods	Modern methods	Traditional methods	Limiting (Female sterilization and male sterilization)	Spacing
	Adjusted relative risk ratio	Adjusted relative risk ratio	Adjusted relative risk ratio	Adjusted relative risk ratio	Adjusted relative risk ratio
Microcredit program (REF: Does not know of any microcredit prog)					
Aware of a program	1.59***	1.45***	1.61***	1.38***	1.55***
	(1.37–1.84)	(1.32–1.59)	(1.39–1.87)	(1.21–1.58)	(1.39–1.74)
Participated in a program	1.32*	1.30***	1.31*	1.16	1.34**
	(0.96–1.80)	(1.09–1.55)	(0.95–1.79)	(0.92–1.46)	(1.05–1.72)
Index-pregnancy related variables					
Parity (REF: 1)					
Parity (2)	1.20**	2.73***	1.01	95.39***	1.13*
	(1.02–1.41)	(2.44–3.06)	(0.86–1.19)	(50.61–179.81)	(0.99–1.28)
Parity (3 or more)	0.99	3.02***	0.85	130.93***	1.04
	(0.81–1.22)	(2.61–3.49)	(0.69–1.03)	(68.50–250.25)	(0.87–1.23)
Son born in any of the births (REF: No)					
Yes	1.03	1.42***	1.00	2.60***	1.08
	(0.88–1.21)	(1.28–1.57)	(0.86–1.17)	(2.18–3.11)	(0.96–1.21)
Gender equity					
Has own money (REF: No)					
	1.16**	1.14***	1.16**	1.15**	1.12**
	(1.01–1.33)	(1.05–1.24)	(1.01–1.32)	(1.02–1.30)	(1.01–1.25)
Freedom of movement (REF: No)					
Yes	1.13*	1.22***	1.13*	1.19***	1.22***
	(0.98–1.29)	(1.11–1.33)	(0.98–1.29)	(1.05–1.35)	(1.09–1.36)
Child marriage (REF: No)					
Yes	0.95	0.92	0.96	1.01	0.90*
	(0.81–1.11)	(0.84–1.02)	(0.82–1.12)	(0.89–1.15)	(0.79–1.01)
Age gap between partners (REF: less than 5)					
5–10 years	1.17*	1.14***	1.17*	1.17**	1.13*
	(0.99–1.38)	(1.04–1.26)	(1.00–1.38)	(1.01–1.35)	(1.00–1.28)
more than 10 years	1.16	1.21**	1.16	1.20	1.22*
	(0.87–1.54)	(1.02–1.43)	(0.87–1.54)	(0.94–1.54)	(0.99–1.51)
Socio-demographics					
Education years (REF: less than 5)					
5–12 years	1.11	1.22***	1.12	1.00	1.51***
	(0.94–1.30)	(1.10–1.36)	(0.95–1.31)	(0.87–1.14)	(1.32–1.74)
more than 12 years	*0.99*	1.21**	1.05	0.74**	1.76***
	(0.76–1.28)	(1.01–1.44)	(0.81–1.36)	(0.56–0.99)	(1.42–2.17)
Religion (REF: Hindu/Other)					
Muslim	0.88	0.88**	0.94	0.38***	1.49***
	(0.74–1.05)	(0.77–0.99)	(0.79–1.13)	(0.31–0.46)	(1.30–1.72)
Caste (REF: Other)					
Caste (OBC)	0.71***	0.72***	0.71***	0.85*	0.69***
	*(0.60 - 0.85)*	*(0.64 - 0.81)*	(0.60–0.85)	(0.72–1.00)	(0.61–0.79)
Caste (SC/ST)	0.82*	0.88**	0.84*	0.92	0.93
	(0.68–1.00)	(0.78–0.99)	(0.69–1.01)	(0.77–1.10)	(0.80–1.07)
Age of woman (continuous)	1.00	0.97***	1.00	0.98***	0.97***
	(0.99–1.02)	(0.96–0.98)	(0.99–1.02)	(0.96–0.99)	(0.95–0.98)
Wealth (REF: 1/Poorest)					
Wealth (2)	1.26**	1.37***	1.24**	1.23**	1.45***
	(1.04–1.53)	(1.21–1.56)	(1.02–1.50)	(1.05–1.45)	(1.22–1.74)
Wealth (3)	1.38***	1.42***	1.34**	1.31***	1.41***
	*(1.10 - 1.73)*	*(1.23 - 1.65)*	(1.07–1.68)	(1.09–1.59)	(1.16–1.71)
Wealth (4)	1.32**	1.41***	1.31**	1.33**	1.49***
	(1.02–1.72)	(1.19–1.66)	(1.01–1.70)	(1.06–1.66)	(1.20–1.85)
Wealth (5/Wealthiest)	1.57***	1.63***	1.58***	1.34*	1.89***
	(1.16–2.12)	(1.34–1.99)	(1.17–2.14)	(0.99–1.80)	(1.48–2.41)
Bank account (REF: does not have a bank account)					
Has a bank account	1.21***	1.09*	1.23***	1.01	1.16***
	(1.06–1.40)	(1.00–1.19)	(1.07–1.41)	(0.89–1.14)	(1.04–1.30)
Access to information (REF: no frequent access)					
Frequent access to information	1.24***	1.60***	1.24***	1.27***	1.72***
	(1.06–1.46)	(1.44–1.79)	(1.05–1.45)	(1.08–1.49)	(1.51–1.96)
Region (REF: North)					
Region (West)	0.36***	0.63***	0.35***	1.49***	0.37***
	(0.27–0.49)	(0.54–0.74)	(0.25–0.47)	(1.21–1.83)	(0.31–0.45)
Region (South)	0.10***	0.57***	0.09***	3.47***	0.11***
	(0.07–0.14)	(0.49–0.65)	(0.07–0.13)	(2.86–4.22)	(0.09–0.13)
Region (Northeast)	1.55***	1.03	1.55***	0.50***	1.18*
	(1.25–1.93)	(0.88–1.21)	(1.25–1.92)	(0.37–0.67)	(1.00–1.40)
Region (East)	0.73***	0.70***	0.72***	0.78**	0.67***
	(0.59–0.90)	(0.61–0.80)	(0.59–0.89)	(0.64–0.95)	(0.57–0.77)
Region (Central)	0.91	0.57***	0.91	0.72***	0.55***
	(0.77–1.07)	(0.51–0.64)	(0.77–1.08)	(0.61–0.85)	(0.48–0.62)
Rural/Urban (REF: Urban)					
Rural	0.87	1.01	0.85*	1.08	0.90
	(0.73–1.03)	(0.90–1.12)	(0.72–1.02)	(0.92–1.26)	(0.79–1.03)

***p < 0.01, **p < 0.05, *p < 0.1.

Results from the models with 'nearness to microcredit programs' as the key predictor showed that living in the same cluster as women who ever took loans from a microcredit program was significantly associated with receipt of minimum four ANC, postnatal care, and use of modern methods of contraceptive postpartum (Supplement Table B).

Even after accounting for self-selection bias using PSM, we observed a significant association of microcredit awareness and participation with receipt of minimum 4 ANC, institutional delivery, postnatal care and use of any modern contraceptives within 12 months of childbirth, supporting an indication of causality (Table 5).

**Table 5:** Adjusted odds ratios (for maternal health outcomes) and adjusted relative risk ratios (for reproductive health outcome) - unmatched and PSM models.

Outcomes	Unmatched	PSM	
		ATE	ATT
Receipt of minimum 4 ANC (AOR) [Reference category: No receipt of minimum 4 ANC]			
Awareness of microcredit program	1.51 (1.40–1.64) ***	1.11 (1.09–1.12) ***	1.10 (1.08–1.12) ***
Participation in microcredit program	1.41 (1.14–1.74) ***	1.09 (1.06–1.13) ***	1.09 (1.06–1.13) ***
Institutional delivery (AOR) [Reference category: Delivery at home]			
Awareness of microcredit program	1.16 (1.05–1.28) ***	1.04 (1.03–1.05) ***	1.04 (1.02–1.05) ***
Participation in microcredit program	1.13 (0.91–1.40)	1.05 (1.02–1.07) ***	1.04 (1.02–1.07) ***
Postnatal care (AOR) [Reference category: No postnatal care]			
Awareness of microcredit program	1.70 (1.57–1.84) ***	1.12 (1.10–1.13) ***	1.11 (1.10–1.12) ***
Participation in microcredit program	1.54 (1.30–1.82) ***	1.09 (1.05–1.12) ***	1.08 (1.05–1.12) ***
Postpartum modern contraceptive use (ARRR) [Reference category: No postpartum contraceptive]			
Traditional method			
Awareness of microcredit program	1.59 (1.37–1.84) ***	1.68 (1.52–1.86) ***	1.69 (1.52–1.87) ***
Participation in microcredit program	1.32 (0.96–1.80)	1.23 (0.92–1.63)	1.23 (0.93–1.63)
Modern method			
Awareness of microcredit program	1.45 (1.32–1.59) ***	1.45 (1.35–1.55) ***	1.45 (1.36–1.55) ***
Participation in microcredit program	1.30 (1.09–1.55) ***	1.28 (1.08–1.52) ***	1.28 (1.08–1.53) ***

***p < 0.01, **p < 0.05, *p < 0.1; Balancing property satisfied at p < 0.05.

To assess differential effects of economic inclusion across wealth status groups, stratified analysis was carried out for all outcomes (Table 6). For women belonging to the lowest quintile of wealth status, both awareness of, and participation in any microcredit program showed significant association with receipt of ANC, institutional delivery and postnatal care. The relationship between program participation and the three outcomes were not significant for the sample of women belonging to the wealthiest group. Use of any modern contraceptive within 12 months of childbirth showed significant association with microcredit program awareness for both lowest and highest wealth quintile.

**Table 6:** Logistic regression models for five wealth quintiles, assessing associations between microcredit programs and receipt of ANC, institutional delivery, postnatal care and postpartum modern contraceptive use.

	Wealth quintile 1 (poorest)	Wealth quintile 2	Wealth quintile 3	Wealth quintile 4	Wealth quintile 5 (wealthiest)
	Adjusted OR	Adjusted OR	Adjusted OR	Adjusted OR	Adjusted OR
Minimum 4 ANC					
Aware of a program	1.58 (1.29–1.92) ***	1.32 (1.09–1.59) ***	1.63 (1.34–1.99) ***	1.49 (1.22–1.81) ***	1.63 (1.28–2.06) ***
Participated in a program	2.32 (1.57–3.42) ***	2.09 (1.44–3.04) ***	1.21 (0.86–1.69)	0.91 (0.50–1.62)	0.70 (0.40–1.22)
Institutional delivery					
Aware of a program	1.22 (1.01–1.04) **	1.17 (0.94–1.46)	1.28 (0.97–1.67)	0.84 (0.59–1.21)	1.15 (0.73–1.79)
Participated in a program	1.69 (1.15–2.48) ***	1.18 (0.73–1.90)	0.87 (0.54–1.39)	1.22 (0.60–2.45)	0.56 (0.13–2.33)
Postnatal check-ups					
Aware of a program	1.63 (1.37–1.95) ***	1.58 (1.32–1.89) ***	1.86 (1.52–2.28) ***	1.81 (1.45–2.24) ***	2.04 (1.55–2.69) ***
Participated in a program	1.67 (1.19–2.35) ***	1.45 (0.98–2.16) *	1.57 (1.11–2.21) ***	2.03 (1.29–3.20) ***	0.86 (0.48–1.55)
Any modern contraceptive within 12 months of childbirth					
Aware of a program	1.40 (1.13–1.73) ***	1.05 (0.86–1.29)	1.43 (1.16–1.77) ***	1.43 (1.16–1.76) ***	1.26 (1.02–1.58) ***
Participated in a program	1.33 (0.87–2.03)	1.33 (0.88–2.01)	1.30 (0.90–1.87)	1.33 (0.92–1.95)	0.79 (0.48–1.31)

***p < 0.01, **p < 0.05, *p < 0.1.

^+^All five models adjusted for all covariates (index-pregnancy related variables, gender equity and socio-demographics).

## Discussion

This study demonstrates a positive association between microcredit programs and health service utilization during and post pregnancy, supporting the relationship between economic approaches to development and women's health. We find that both awareness of and participation in microcredit programs are associated with the four assessed indicators of health service utilization. These relationships hold true even after accounting for self-selection bias. Our study extends prior research from India that has indicated a positive relationship between aspects of financial inclusion such as bank account ownership and women's health (Saggurti et al., 2018; Singh et al., 2019, p. 100396) by examining financial participation, via microcredit programs. Even after controlling for bank account ownership and other economic factors such as wealth status, we find that microcredit programs have a strong relationship with use of health services during and post pregnancy. As financial inclusion of women increases globally as well as in India (Demirguc-Kunt & Klapper, 2012), greater coverage of financial participation through microcredit programs may lead to higher returns with respect to women's maternal and reproductive health.

The relationship between microcredit program participation and increased health service utilization can be explained by a number of potential pathways. They may include increased ability to pay for health services (Braveman et al., 2010) and increased information on health services as a result of creation of social networks by the programs (Ruducha et al., 2018). The associations of microcredit program awareness, or of living close to a microcredit program participant, with maternal health service utilization could be indicative of a spillover effect. Non-participants who live near microcredit programs may benefit from the programs, as a result of creation of social networks for spread of health-related information. This can be particularly true for microcredit programs which follow a group lending or SHG model where members meet and engage with each other regularly. Studies argue that social capital is an inherent aspect of SHGs, which then functions to create opportunities to access social support and information (Anderson, Locker, & Nugent, 2002; Sanyal, 2009). Additionally, there has been a steady growth of SHG-led health interventions in India, which aim to improve maternal, child and reproductive health outcomes (UNICEF, 2012); the majority of these programs provide members with information on required pregnancy care, postnatal care and child care and importance of contraceptive use. As cited in Banerjee et al., participants of microcredit programs are likely to pass on health-related information to their friends and acquaintances (A. Banerjee, Chandrasekhar, Duflo, & Jackson, 2013), potentially leading to improved use of health services among non-members in the community. Regular meetings with the members of the microcredit program can also make women more adept at communicating and expand their social networks. If a microcredit program or SHG actively promotes health-related programs, that promotion may also increase demand within the community for the necessary services. Further qualitative research to understand mechanisms by which nearness to microcredit programs can lead to improved maternal and reproductive health are required. By implementing microcredit programs in different parts of India, especially the ones that follow a model which allows transfer of information among non-participants, or creation of social networks, use of maternal and reproductive health services can be improved.

In line with prior studies, we find a distinct economic and social divide between participants and non-participants (Schuler & Hashemi, 1994; van Ophem & Antonides, 2016). Microcredit program participants differ from non-participants in terms of education, caste, religion, parity, child marriage and freedom of movement. In terms of geography, women residing in southern states of the country are more likely to be aware of and participate in a microcredit program. These results are not surprising, given that around 70% of SHGs in India are in four southern states of the country (Deininger & Liu, 2009).

Our findings suggest that it is the wealthier women who are more likely to be aware of and participate in microcredit programs. However, microcredit programs were developed to target poor women, who are in need of credit to engage in income generating activities. Interestingly, wealth quintile-specific analysis show that the health effects are consistently significant only for the poorest group of women, perhaps as they have the greatest gaps in health service utilization coverage (International Institute Population Sciences and ICF). This points to the need for strengthened efforts to expand the coverage of these programs among the economically marginalized population. Poorer households have a greater lack of economic and social capital which prevents them from accessing important maternal and reproductive health care. Microcredit programs can help bridge this gap by providing them with credit as well as creating social networks and social support.

We find a positive association between microcredit program participation and use of postpartum modern contraceptive. With respect to method type, we observe a significant relationship with use of spacing methods. This has important programmatic implications. Women participating in microcredit programs, and consequently, in economically productive activities, might desire greater flexibility over their own reproductive health, which is provided by spacing methods. This is consistent with prior research from India which shows that women's access to money is associated with use of oral contraceptive pills and condoms (Reed et al., 2016). However, female sterilization continues to dominate contraceptive use in India. Our findings are particularly important in the context of interventions that use microcredit programs or SHGs as a platform to improve awareness, and subsequently, use of contraceptives. Further studies are required to understand the pathways between microcredit programs and specific behavior related to use of different contraceptive methods.

### Limitations

This study has several important limitations. First, these data are cross-sectional and lacks temporality, so we cannot make direct inferences on causality. However, by using IPTW as a matching technique to account for selection bias, and the positive results from the models of maternal health utilization for the sub-sample of women who gave birth in the last year, our study does indicate a potential causal relationship. Second, the data did not include detailed information about the activities that were implemented by the microcredit programs. As a result, it remains unclear which of the various microcredit models (group lending or SHG, SHG integrated with health program, microfinance program) are most effective. However, given that more than 80% of the microcredit programs in India are reported to follow the SHG structure, our findings may have greater meaning in the context of the group-lending models. Next, while our study accounted for self-selection bias by matching for individual-level characteristics, we did not account for any community/village level characteristics due to unavailability of data. Previous studies have observed positive effects of village-level factors such as presence of a health facility, distance of health facility from the village, and local governance on women and child's utilization of health services (Dhak, 2013; Hamal, de Cock Buning, De Brouwere, Bardají, & Dieleman, 2018; Nair & Panda, 2011). Additionally, with respect to presence of microcredit programs in a village, some communities might be more welcoming towards setting up of such institutions, owing to prevalent social norms. The number of microcredit programs present within a village might also have an effect on the relationship between the programs and the concerned indicators of health. Further studies which focus on both the individual and village level indicators could provide more insights on this topic of interest. Fourth, the study does not capture any information on membership of microcredit programs, limiting the analysis to awareness, and participation in the form of borrowing money from the programs. Information on timing of membership and frequency of meetings by program members, aspects which have been noted as important elsewhere (Hamad & Fernald, 2015), were also not collected. Next, while our study provides population level effect sizes for microcredit programs in India, it is important to note that there might be individual observations of negative impacts, owing to women being trapped in a cycle of debt as indicated by a few studies (Rahman, 1999). Further qualitative research can investigate the presence of such negative impacts. Finally, the study is specific to India and findings are not generalizable to other geographies. However, they do offer insights into similar settings which has a majority of microcredit programs functioning as SHGs.

## Conclusion

The current study documents the association between microcredit programs and maternal and reproductive health service utilization in India. It suggests that the health effects are not limited to the participants alone; non-participants in the community who are aware of the programs also have higher odds of accessing these services. Findings highlight the important role of financial participation in achieving improved health, particularly among poor women, for whom effects were most robust. Further research should explore the causal pathways through which these associations may occur, especially with respect to observed effects among non-participants. In contexts such as India where wealth differentials in maternal health service utilization remain pronounced, addressing the economic status of women by providing them with access to credit could prove to be an effective intervention.

## Ethics approval

The paper uses National Family Health Survey - 4 data, a publicly available dataset with no identifiable information. No additional data were gathered from any human subjects for the purpose of this study.

## Conflicts of interest

None.

## Financial disclosure statement

This study was supported by the Bill & Melinda Gates Foundation [OPP1179208]; and National Institutes of Health [R01HD084453-02].
